# Cortico-Hippocampal Brain Connectivity-Guided Repetitive Transcranial Magnetic Stimulation Enhances Face-Cued Word-Based Associative Memory in the Short Term

**DOI:** 10.3389/fnhum.2020.541791

**Published:** 2020-10-30

**Authors:** He Wang, Jingna Jin, Dong Cui, Xin Wang, Ying Li, Zhipeng Liu, Tao Yin

**Affiliations:** ^1^Institute of Biomedical Engineering, Chinese Academy of Medical Science and Peking Union Medical College, Tianjin, China; ^2^Neuroscience Center, Chinese Academy of Medical Science and Peking Union Medical College, Beijing, China

**Keywords:** cortico-hippocampal connectivity, rTMS (repetitive transcranial magnetic stimulation), associative memory, target location, precise navigation

## Abstract

Repetitive transcranial magnetic stimulation (rTMS) can be used to enhance the associative memory of healthy subjects and patients with Alzheimer’s disease (AD). However, the question of where the stimulation should be applied is still unresolved. In a preliminary survey for an effective and feasible solution to this problem, we identified three representative rTMS targets using cortico-hippocampal connectivity, calculated using resting-state fMRI (rs-fMRI) data from 80 young, healthy subjects: (1) the cortical area with the strongest connectivity across the whole cerebral cortical area; (2) the whole lateral parietal cortical area; and (3) the whole medial prefrontal cortical area. We then compared the short-term effects on associative memory, which was tested using face-cued word recall by applying rTMS to three identified targets in a single population of eight healthy adults. Each treatment lasted for 2 days. Associative memory performance was measured at four time points: before and after stimulation on the first day (baseline and post 1) and before and after stimulation on the second day (post 2 and post 3). Compared with baseline levels, 20 min of high-frequency rTMS delivered to target 2 or target 3 produced a significant increase in the mean accuracy of associative memory performance at the post 3 time point alone (target 2, *P* = 0.0035; target 3, *P* = 0.0012). Compared with the sham conditions, significant increases in the mean associative memory performance were observed when high-frequency rTMS was delivered to target 2 (*P* = 0.02) and target 3 (*P* = 0.012), but not when delivered to target 1 (*P* = 0.1). Compared with baseline levels, 20 min of high-frequency rTMS delivered to target 3 produced a significant reduction in the mean reaction time of associative memory only at time points post 1 (*P* = 0.0464) and post 3 (*P* = 0.0477). Compared with the sham conditions, significant reductions in the mean reaction time of associative memory were observed when high-frequency rTMS was delivered to target 3 (*P* = 0.006), but not when delivered to target 1 (*P* = 0.471) or target 2 (*P* = 0.365). Our findings indicate that stimulation of the locations with the strongest cortico-hippocampal connectivity within the lateral parietal cortical or medial prefrontal cortical areas is effective in enhancing face–word recall-based associative memory in the short term.

## Introduction

The hippocampus plays an important role in associative memory (Gordon, 2011; Preston and Eichenbaum, 2013). Neurodegenerative pathological changes in the hippocampus may lead to Alzheimer’s disease (AD), which is characterized by a progressive loss of associative memory (Frisoni et al., 2009; Ballard et al., 2011). At present, the cause of AD is still not clearly understood, but numerous studies have demonstrated that brain atrophy and reduced neuroplasticity are two major causes of the disease. Repetitive transcranial magnetic stimulation (rTMS) can be used to create a current in the human brain using a pulsed magnetic field; this current is able to change the neuronal excitability to facilitate neuroplasticity (Rabey et al., 2011, 2013; Zhang et al., 2019). For this reason, rTMS has been employed as a non-invasive and painless physical therapy for patients with AD.

The hippocampus is located deep within the human brain. Traditional rTMS coils are unable to stimulate deep regions such as the hippocampal area in the brain; however, with deep rTMS coils, this is possible but with poor focality (Bible, 2012). Therefore, traditional rTMS coils have been widely used to stimulate the cerebral cortical area associated with the hippocampus, which indirectly regulates the excitability of the hippocampus to improve associative memory in patients with AD (Cheng et al., 2018).

Recent studies have demonstrated that rTMS can significantly enhance the long-term acquisition of associative memory in patients with AD (Cotelli et al., 2006). Previous studies have also shown that stimulation of the dorsolateral prefrontal cortex (DLPFC), which is known to be a node of the central-executive network, using a high-frequency rTMS pulse, can enhance the long-term retrieval of associative memory (Kumar et al., 2017a). In patients with AD, studies have shown that the loss of plasticity in the DLPFC is related to the loss of retrieval of associative memory and word comprehension. Therefore, stimulating the DLPFC to increase the excitability of this region could alleviate such symptoms (Kumar et al., 2017b).

Moreover, the DLPFC is a Food and Drug Administration-approved target for the treatment of major depressive disorder (Mantovani et al., 2007). Considering that a significant proportion of patients with AD have depressive symptoms, whether the effects of rTMS in patients with AD are due to a reduction in depressive symptoms or an enhancement of short-term retrieval of associative memory remains controversial (Starkstein et al., 2005; Rutherford et al., 2013). Additionally, the parietal lobe, which is part of the parietal–hippocampal circuit, is another effective target for rTMS (Wang et al., 2014). Zhao et al. (2016) showed that high-frequency rTMS of the parietal lobe enhances cognition in patients with AD.

Stimulation of the right inferior frontal gyrus or the superior temporal gyrus with rTMS is also effective in improving performance on attention-related tests (Anderkova et al., 2015). Furthermore, a previous study has demonstrated that high-frequency rTMS of the dorsal precuneus can also enhance the short-term retrieval of associative memory and improve precuneal–hippocampal connectivity (Koch et al., 2017). Overall, previous studies have shown that stimulating cortical regions connected to the hippocampus can improve cognition in patients with AD. However, at present, it is not clear which brain regions are the most effective targets for enhancing associative memory.

Neuroanatomical studies indicate that the hippocampus processes information gathered from distributed cortical areas and combines these different streams of information into associative memories (Parent et al., 2010). Current understanding from related research shows that the interaction between the hippocampus and distributed cortical areas plays an important role in the retrieval of associative memory (Titone et al., 2004; Ranganath and Ritchey, 2012). For instance, the functional connectivity between the frontal lobe and the hippocampus has been proven to relate to the retrieval of associative memory for both facts and sources (Fan et al., 2000). The impairment in the network between the left inferior parietal lobe and the left hippocampus damages the episodic memory in mild cognitive impairment patients (Christiane et al., 2011). Besides that, some studies have proved that applying rTMS on the dorsolateral prefrontal cortex and the parietal cortex can significantly improve the retrieval of associative memory function in healthy subject (Turriziani et al., 2012; Li et al., 2017). A number of previous studies have used functional magnetic resonance imaging (fMRI) to locate distributed cortical areas connected to the hippocampus and have demonstrated that these areas are related to the short-term retrieval of associative memory (Wang et al., 2014). Based on these findings, we hypothesized that, by locating target regions based on cortico-hippocampal brain connectivity from fMRI data, we could target the same brain regions with rTMS to enhance associative memory and identify which are the most effective targets.

In the present study, we therefore used the Human Connectome Project (HCP; Van Essen et al., 2013) rs-fMRI image dataset, collected from 80 young, healthy subjects to obtain cortico-hippocampal connectivity information. We then analyzed these data to identify potentially effective targets for associative memory enhancement. Two parcels were defined using the left and the right hippocampal masks from the automated anatomical labeling (AAL) template. The cerebral cortices of the left and the right hemispheres were each segmented into 500 parcels using the brain atlas published by Schaefer et al. (2017). The cortico-hippocampal connectivities between the segmented cortical parcels and the hippocampal parcels were calculated using Granger causality analysis (GCA).

Three representative parcels which showed significant functional connectivity with the hippocampus in the corresponding hemisphere were selected as stimulation targets for rTMS. Eight subjects were recruited for the rTMS and memory experiments. For two consecutive days, 10 Hz of rTMS was applied to the targets, and face-cued word recall tests were used to measure changes in associative memory, both before and after stimulation in the short term. A sham control rTMS experiment was carried out using similar parameters; however, the stimulation was applied by tilting the coil to 90° relative to the scalp, with the two wings of the coil touching the scalp.

The aim of the present study was to compare the efficacy of different cortical locations identified from cortico-hippocampal connectivity data. Identifying the most effective areas for stimulation to enhance associative memory would make a significant contribution to the rTMS treatment of patients with AD.

## Materials and Methods

### Subjects and rs-fMRI Dataset

Eight healthy subjects (five females and three males, aged 23–34 years) were recruited for the rTMS experiments. All subjects were right-handed, and no signs of discomfort were observed throughout the study. The experimental procedures were approved by the Joint Ethics Committee of the Chinese Academy of Medical Sciences and Peking Union College. All experiments were performed according to the principles of the Declaration of Helsinki. All study participants had normal or corrected-to-normal vision. None of the participants were diagnosed with any psychiatric disease; neither were any taking any psychoactive medications. Written informed consent was obtained from all subjects, who were paid for their participation in the experiment.

The rs-fMRI and T1 data of the first 80 participants from the HCP database were analyzed in the present study (Van Essen et al., 2013). The image data were collected on a 3T Skyra scanner (Siemens, Erlangen, Germany) with a 32-channel head coil. The blood oxygenation level-dependent (BOLD) signals were acquired with a gradient-echo echo-planar imaging sequence, using the parameters defined in the HCP protocols. Four runs of rs-fMRI were obtained with the subjects’ eyes open and relaxed fixation on a bright, projected cross-hair on a dark background.

In total, 1,200 frames were acquired in each run, which lasted 14:33 (min:s). The oblique axial acquisitions alternated between phase encoding in a right-to-left (RL) direction in one run and phase encoding in a left-to-right (LR) direction in the other run. The first two runs of the rs-fMRI data were selected for further analysis. The T1 images were collected with a magnetization-prepared rapid gradient-echo sequence, using the parameters defined in the HCP protocols.

### rs-fMRI Preprocessing

The rs-fMRI and T1 data were preprocessed using the Statistical Parametric Mapping 12 toolbox (SPM12; London, UK) in the MATLAB vs. 2013b software (MathWorks, Natick, MA, USA). The rs-fMRI volumes were realigned to the mean volume of each run. The T1 volumes were co-registered to realigned rs-fMRI volumes. All volumes were then normalized to the Montreal Neurological Institute (MNI) space using the standard segmentation method. The rs-fMRI volumes were then smoothed with an isotropic Gaussian kernel of 4 mm full width at half-maximum.

### Definition of Regions of Interest

The cerebral cortex of the rs-fMRI volumes was parceled into 1,000 regions of interest (ROIs) using the brain atlas published by Schaefer et al. (2017). A total of 500 ROIs were acquired from each of the left and the right hemispheres. Two ROIs were defined using the left and the right hippocampus masks of the AAL template, as reported in previous studies.

### GCA-Based Cortico-Hippocampal Brain Connectivity

The GCA was applied to identify valid connections between the time series of target ROIs (for both the right and the left hippocampi) and the time series of each ROI within the right and the left cerebral cortex. The ROI-wise GCA was conducted using the rsHRF in the SPM12 toolbox. Originally, the GCA algorithm was used in economics to assess causal relationships between two data sequences (Zang et al., 2012). The essential idea of the algorithm suggests that if the past of one data sequence (the “cause”) is useful to predict the future of the other data sequence (the “result”), then effective connectivity exists between the two data sequences.

In the current study, the time series of ROIs of the left and the right hippocampi were defined as *y*, and the time series of ROIs within the right and the left cerebral cortices were defined as *x*. The linear direct connectivities of *x* to *y* (C-to-H) were determined using ROIs from across the whole cerebral cortex. The mean connectivities were calculated using the first two runs of the rs-fMRI data to identify the target rTMS locations.

### Identification of Stimulation Targets

The stimulation targets were identified using group-level hippocampal resting-state functional connectivity obtained from the first 80 subjects of the HCP dataset. The T1 data for each subject were transformed to the MNI space using the SPM12 toolbox. The transformation matrix was preserved to apply the transformation between the MNI space and the original space. Based on the 1,000 connections from the cortex to the hippocampus in the dataset, three representative targets were selected as stimulation locations: (1) the location with the strongest connectivity across the whole cerebral cortical area (target 1); (2) the location with the strongest connectivity across the whole lateral parietal cortical area (target 2); and (3) the location with the strongest connectivity across the whole medial prefrontal cortical area (target 3).

### rTMS Parameters and Procedure

We delivered high-frequency rTMS to the stimulation targets on two treatment days, using a Magstim Rapid stimulator (Magstim, Whitland, UK), and an air-cooled, figure-of-eight coil, TMS Neuronavigation tool (Brainsight, Montreal, Quebec, Canada). The stimulation location that continuously generated the strongest motor-evoked potentials (MEPs) at the right first dorsal interosseus (FDI) muscle was identified on the cerebral cortex of each subject. The minimum stimulation output that was able to create a MEP larger than 50 mV in at least five out of 10 pulses at the right FDI muscle was selected as the resting motor threshold (RMT).

**Figure 1:**
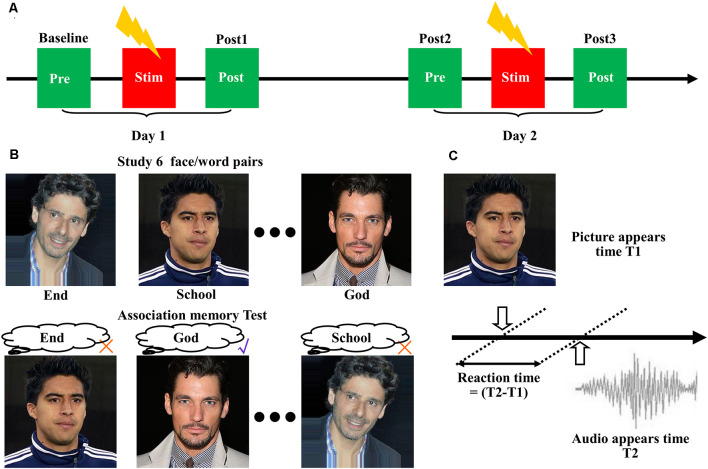
Cortico-hippocampal brain connectivity-guided repetitive transcranial magnetic stimulation (rTMS) improved associative memory. **(A)** Experimental procedures. The subjects received four treatments at the three stimulation targets, plus one sham stimulation. Each treatment lasted for 2 days, and associative memory performance was measured at the following time points: before stimulation on the first day (baseline), after stimulation on the first day (post 1), before stimulation on the second day (post 2), and after stimulation on the second day (post 3). Each pair of treatments was separated by at least 5 days to reduce the after-effects. **(B)** Design of the face-cued word recall measure of associative memory. **(C)** Schematic representation of reaction time extraction.

A single session of 20-min high-frequency (10 Hz) stimulation (100% RMT) was delivered to the three identified targets using a figure-of-eight coil, with each block lasting 5 s and being repeated every 30 s. For comparison, we performed a sham experiment by tilting the coil at 90° relative to the scalp, with the two wings of the coil touching the scalp. All eight subjects received four treatments at the three different stimulation targets, plus one sham stimulation. Figure 1A shows each treatment lasting for 2 days. Associative memory performance was measured at the following time points: (1) before stimulation on the first day (baseline); and (2) after stimulation on the first day (post 1), before stimulation on the second day (post 2), and after stimulation on the second day (post 3). Each pair of treatments was separated by at least 5 days to reduce the after-effects.

### Associative Memory Measurement

We used a face-cued word recall test to measure associative memory performance (Figure 1B). Each subject studied six images of human faces, each of which was presented separately and in succession on a black background for 3 s. A noun (single word) was placed under each picture. The subjects were instructed to remember the association between the picture and the noun. After a 30-s delay, the same six pictures were presented separately to the subject in random order without the words. The subjects were asked to recall the associated words. No feedback was given to the participants about the correctness of their response. Five separate measurements with different pictures and words were performed according to a Latin square design.

The accuracy of associative memory performance was defined as the percentage correct out of 30. The reaction time was determined based on the presentation time of the images and the audio (Figure 1C). The images of the faces were selected from the IMDB-WIKI dataset^1^ (Rothe et al., 2018). The words were selected from the MRC Psycholinguistic Database^2^. The words were three to nine letters in length and were based on Kucera–Francis frequencies of 300–2,000 and concreteness ratings of 200–800. To remove the influence of individual differences, all subsequent statistical analyses were performed using the changes relative to baseline.

### Data Analysis

Because of the low signal-to-noise ratio (SNR) of the BOLD signals, two-tailed, paired *t*-tests were used to evaluate the differences between the two runs (LR direction and RL direction). Analyses were conducted in terms of the cortico-hippocampal brain connectivities of the 1,000 ROIs. Levene’s test was applied to test the homogeneity of variance of the behavioral experiment results. A two-way repeated-measures analysis of variance (ANOVA) was applied to analyze associative memory performance. The factors considered in the two-way repeated-measures ANOVA were the four stimulation targets (sham, target 1, target 2, and target 3) and the four time points (baseline, post 1, post 2, and post 3) at which associative memory performance was measured. Tukey’s correction method was used to perform multiple-comparison *post-hoc* *t*-tests for all tests and all stimulated regions. All data analyses were performed on IBM SPSS Statistics, version 21 (IBM Corporation, Armonk, NY, USA).

## Results

### Stability of Cortico-Hippocampal Connectivity

For each subject, rs-fMRI data were collected from two runs. These runs comprised oblique axial acquisitions alternating between phase encoding in a RL direction for one run and phase encoding in a LR direction for the next run. Considering the direction of connectivity, four cortico-hippocampal connectivity values were obtained for each cortical ROI of each subject.

One of the disadvantages of the BOLD signal is the relatively low values of the SNR. Therefore, we tested differences using cortico-hippocampal connectivity values calculated from rs-fMRI data acquired during two runs. Two-tailed, paired *t*-tests revealed no significant differences between the two runs for 840 out of 1,000 connections from the cortex to the hippocampus. No significant differences were observed between the two runs for 815 out of 1,000 connections from the hippocampus to the cortex.

### Stimulation Target Identification

**Figure 2:**
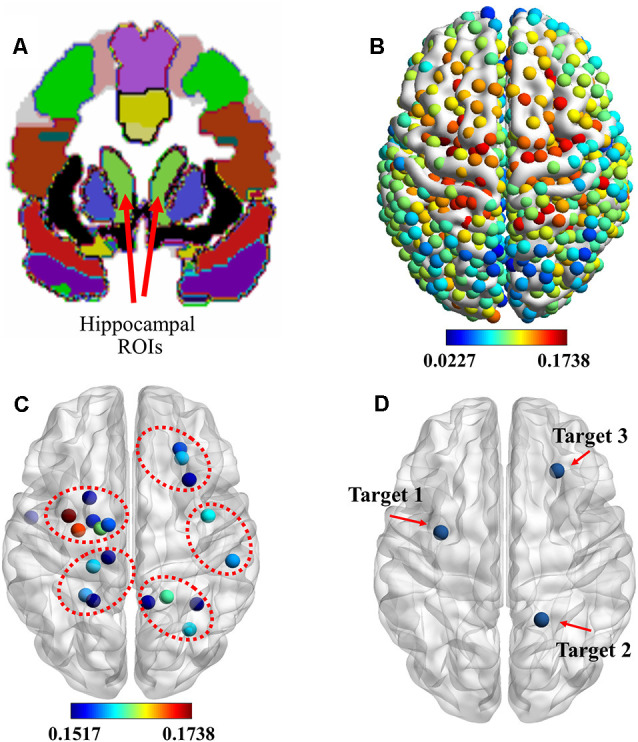
Identification of stimulation targets. **(A)** Two light-green hippocampal regions of interest (ROIs) selected from the automated anatomical labeling (AAL) template in the Montreal Neurological Institute (MNI) space. **(B)** ROIs showing the functional magnetic resonance imaging (fMRI) connectivity of the cortex with the hippocampal ROI. **(C)** Top 20 ROIs showing the fMRI connectivity of the cortex with the hippocampus. **(D)** Three stimulation targets were located based on the maximum fMRI connectivity of the cortex with the hippocampus from the whole cerebral cortical, lateral parietal cortical, and medial prefrontal cortical areas. rTMS was delivered to these targets under the guidance of a neuronavigation system.

Based on previous findings (Wang et al., 2014), the cortico-hippocampal connection of C-to-H should be selected to identify the location of stimulation targets for rTMS. Figure 2A shows the hippocampal ROIs that were selected from the AAL template in the MNI space. The connectivity of the cortex to the hippocampus, as indicated by the means of the GCA connectivity in 80 subjects, is presented in Figure 2B. In agreement with previous findings, we observed that the ROIs with more effective connectivity existed in parts of the prefrontal and the parietal lobes. To illustrate more precise positioning of the effective areas, the 20 ROIs with the strongest connectivities were determined and are presented in Figure 2C.

These data show that the hippocampus was effectively connected to ROIs near the superior parietal gyrus, paracentral lobule, precentral gyrus, and dorsolateral superior frontal gyrus. Based on these results, combined with the results of previous research, we selected three targets, which are presented in Figure 2D. They were: (1) target 1, located at the position with the strongest connectivity across the whole cerebral cortical area; (2) target 2, located at the position with the strongest connectivity across the whole lateral parietal cortical area; and (3) target 3, located at the position with the strongest connectivity across the whole medial prefrontal cortical area. Detailed information on the three targets is presented in Table 1.

**Table 1:** Information on the three stimulation targets selected with cortico-hippocampal brain connectivity.

Granger causality analysis (GCA) rank	GCA connectivity	Montreal Neurological Institute (MNI) coordinate (mm)			Lobe	Gyrus
		*X*	*Y*	*Z*		
1	0.1738	−33	−3	51	Frontal lobe	Middle frontal gyrus
4	0.1615	19	−48	70	Parietal lobe	Superior parietal lobule
8	0.1582	27	29	42	Frontal lobe	Middle frontal gyrus

### Changes in Associative Memory Performance

**Figure 3:**
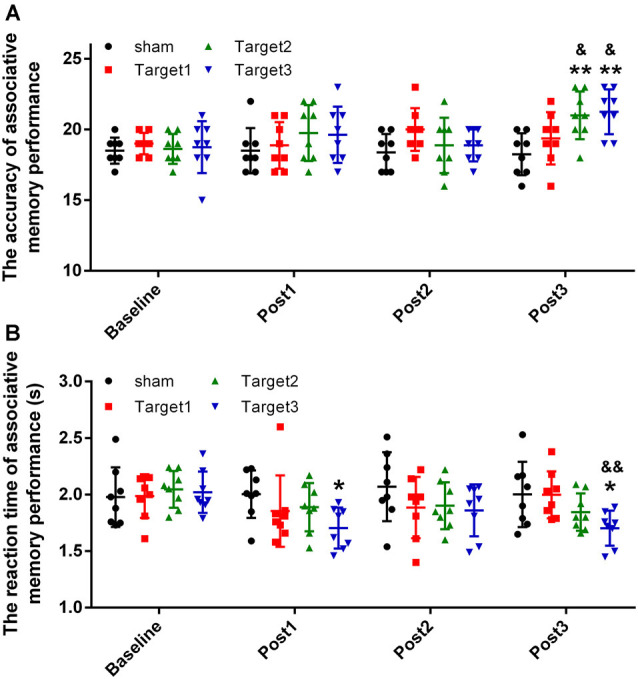
Experimental results. **(A)** Stimulation-induced changes in the mean accuracy of associative memory performance. **(B)** Stimulation-induced changes in the reaction time of associative memory performance. Data are expressed as mean ± standard deviation. *P < 0.05, **P < 0.01 vs. baseline group; ^&^*P* < 0.05, ^&&^*P* < 0.01 vs. sham group.

The short-term effects on face-cued word-based associative memory performance after stimulation of each target are detailed in Figure 3A (accuracy) and Figure 3B (reaction time). Levene’s test revealed that the experimental data satisfy the homogeneity of variance (accuracy, *P* = 0.226; reaction time, *P* = 0.886). In terms of the accuracy of associative memory performance, a two-way ANOVA revealed a significant effect of the stimulation target [*F*_(3,112)_ = 4.149; *P* = 0.008; sum-of-squares (SS) = 30.461; observed power (OP) = 0.841] and time point (*F*_(3,112)_ = 3.697; *P* = 0.014; SS = 27.148; OP = 0.792). For the reaction time, a two-way ANOVA showed a significant effect of stimulation target (*F*_(3,112)_ = 3.807; *P* = 0.012; SS = 0.591; OP = 0.805), but no significant effects of time point (*F*_(3,112)_ = 2.505; *P* = 0.063; SS = 0.389; OP = 0.607). No significant interactions were noted between the stimulation target and the time point for either accuracy (*F*_(9,112)_ = 1.757; *P* = 0.084; SS = 38.695; OP = 0.768) or reaction time (*F*_(9,112)_ = 1.09; *P* = 0.376; SS = 0.508; OP = 0.517).

The accuracy measurements obtained at the three time points after stimulation were compared with baseline values, using Tukey’s multiple-comparisons test. Compared with baseline, 20 min of high-frequency rTMS delivered to target 2 and target 3 produced a significant increase in the mean accuracy of associative memory at post 3 time point alone (target 2, *P* = 0.0035; target 3, *P* = 0.0012). However, no significant changes were observed either under sham conditions or when target 1 was stimulated at any of the three time points. Compared with the sham conditions, significant increases in the mean accuracy of associative memory were observed when high-frequency rTMS was delivered to target 2 (*P* = 0.02) and target 3 (*P* = 0.012), but not when delivered to target 1 (*P* = 0.1). No significant changes were observed in the mean accuracy of associative memory when target 2 was compared with target 3 (*P* = 0.999).

The reaction times obtained at the three time points after stimulation were compared with baseline values using Tukey’s multiple-comparisons test. Compared with baseline, 20 min of high-frequency rTMS delivered to target 3 produced a significant reduction in the mean reaction time of associative memory at time points post 1 (*P* = 0.0464) and post 3 (*P* = 0.0477) alone. No significant changes were observed under sham conditions nor when target 1 or target 2 were stimulated at any of the three time points. Compared with the sham conditions, significant reductions in the mean reaction time of associative memory were observed when high-frequency rTMS was delivered to target 3 (*P* = 0.006), but not when delivered to target 1 (*P* = 0.471) or target 2 (*P* = 0.365). No significant changes in the mean reaction time of associative memory were observed when target 2 was compared with target 3 (*P* = 0.310).

## Discussion

In the present study, we conducted cortico-hippocampal brain connectivity-guided rTMS to enhance associative memory in healthy subjects. Three cortical targets were identified using GCA between the cortex and the hippocampus, based on rs-fMRI data from 80 subjects in the HCP project. The short-term effects of stimulating the three targets on face-cued word-based associative memory were compared in eight healthy subjects. The results indicate several important findings. First, significant increases in accuracy and reductions in reaction time of associative memory were noted following stimulation of target 3. This target was located at the position with the strongest connectivity across the whole medial prefrontal cortical area. Second, the only significant increases in the accuracy of associative memory were observed in target 2. This target was located at the position with the strongest connectivity across the whole lateral parietal cortical area. Third, even though target 1 was the most connected cortical location, no significant changes were observed in the accuracy or reaction time following stimulation. These findings all suggest that the cortico-hippocampal brain connectivity of the lateral parietal cortical area and the medial prefrontal cortical area can be used to locate targets to enhance associative memory in healthy subjects.

At present, the underlying reason for dysfunctions in neuroplasticity, which result from pathological changes in AD, remain unclear. There may be an association between the plasticity of the cerebral cortex and the pathological features of AD (Frisoni et al., 2009; Ballard et al., 2011). Additionally, synaptic dysfunction could result in a malignant cycle of anomalous neuroplasticity and beta-amyloid deposition, leading to further deterioration in AD (Labar et al., 1999). However, some studies have demonstrated a positive relationship between low levels of amyloid and the preservation of neuroplasticity (Morley and Farr, 2012). Damage to functional networks has been widely investigated and is reportedly related to increased amyloid deposition and decreased cognitive ability (Wang et al., 2006). The central-executive and the hippocampal–parietal networks are two typical representatives of such functional networks (Wang et al., 2014). In the present study, we determined two effective target locations in the MNI space on the cortex to enhance associative memory in the short term. One effective target was located on the lateral parietal cortical area, and the other effective target was located on the medial prefrontal cortical area. The present findings are consistent with those of previous studies. The target locations were calculated based on rs-fMRI data from 80 subjects in the HCP project, and the targets were then applied to eight healthy subjects in the present study. Therefore, the effectiveness of the targets should be proved on a greater number of subjects in future studies.

As a key node of the central-executive network in the cortex, the DLPFC is the most widely used stimulation target to treat patients with AD (Cheng et al., 2018). Some studies have shown that the cognitive scores, working memory, and language comprehension of patients with AD are significantly improved by rTMS intervention at the DLPFC (Cotelli et al., 2006; Kumar et al., 2017a). Additionally, the parietal cortex, a node of the hippocampal–parietal network, has been targeted, and a significant improvement of cognition was observed in patients with mild AD (Wang et al., 2014). Furthermore, delivering rTMS to the right inferior frontal gyrus improves attention and cognitive speed in patients with AD (Anderkova et al., 2015). In healthy subjects, rTMS increases neural plasticity and enhances brain networks (Wang et al., 2014; Zhao et al., 2016). Determining the optimal brain regions for the enhancement of associative memory will be beneficial for the clinical application of rTMS in patients with AD (Heath et al., 2018). Therefore, in contrast to previous studies, in the present study, corresponding changes in associative memory were evaluated for three targets. Behavioral experiments were used to identify which targets were more effective. We used cortico-hippocampal connectivity analysis to identify several ROIs in the prefrontal and the parietal lobes that were strongly connected with the hippocampus. The results are consistent with those of previous studies (Cotelli et al., 2006; Rutherford et al., 2013; Wang et al., 2014; Zhao et al., 2016; Cheng et al., 2018). Furthermore, we selected three representative ROIs for rTMS stimulation in healthy subjects. Our results indicate that targets in the medial prefrontal cortical area and the lateral parietal cortical area are more effective at modulating the accuracy of face-cued word-based associative memory in the short term. However, no significant differences in associative memory were observed between these two targets. Targeting the medial prefrontal cortical area alone improved the reaction time of associative memory recall. In contrast to the lateral parietal cortex, the medial prefrontal cortex was involved in many other brain functions, including mood regulation (Lyketsos et al., 2003). This likely explains why target 3 had a greater effect than target 2 on the reaction time. To understand the ineffectiveness of target 1, the major functions of the closest functional region in the Human Brainnetome Atlas, labeled A6vl, were investigated. They were identified as executive action, motor learning, and spatial cognition (Lingzhong et al., 2016). This region had good connectivity with the hippocampus, but no effects on associative memory were observed following its stimulation. In future studies, we can test whether stimulation of target 1 can effectively improve motor learning. Furthermore, it will be interesting to clarify whether the simultaneous stimulation of target 2 and target 3 produces a more effective modulation of associative memory.

Both the rs-fMRI data and the behavioral data were obtained from healthy adults. Thus, the findings of the present study are not directly applicable to patients with AD. Additional experiments should be conducted on subjects with the disease to verify the results of the present study. In a number of previous studies, both short-term and long-term rTMS were able to improve the cognitive scores in patients with AD. Some studies have indicated that the long-term stimulation paradigm yields better effects (Cotelli et al., 2006; Kumar et al., 2017a,b; Cheng et al., 2018). As only 2 days of rTMS was applied in this study, a long-term stimulation protocol may produce better results and should be evaluated in the future. Furthermore, associative memory performance was measured after a relatively short time (less than 20 min). The long-term effects of rTMS on these targets will also need to be evaluated in future studies.

Previous studies have shown ample evidence that rTMS improves associative memory. Therefore, to maximize these benefits, we located cortical targets based on cortico-hippocampal connectivity analysis. We compared the short-term effects of stimulating three representative targets in a single group of subjects. Our results indicate that stimulating the location with the strongest cortico-hippocampal connectivity in the lateral parietal cortical area or the medial prefrontal cortical area can effectively improve the accuracy of face-cued word-based associative memory in the short term. In addition, our results show that the reaction time can be improved by stimulating the location with the strongest cortico-hippocampal connectivity in the medial prefrontal cortical area.

## Data Availability Statement

The datasets generated for this study are available on request to the corresponding author.

## Ethics Statement

The studies involving human participants were reviewed and approved by Joint Ethics Committee of the Chinese Academy of Medical Sciences and Peking Union College. The patients/participants provided their written informed consent to participate in this study.

## Author Contributions

HW, ZL and TY contributed to the conception and design of the study. HW organized the data collection. JJ performed the statistical analysis. XW and YL organized the experiment. HW wrote the first draft of the manuscript. All authors contributed to the article and approved the submitted version.

## Conflict of Interest

The authors declare that the research was conducted in the absence of any commercial or financial relationships that could be construed as a potential conflict of interest.

## Footnotes

^1^https://data.vision.ee.ethz.ch/cvl/rrothe/imdb-wiki/

^2^http://www.psych.rl.ac.uk
